# Profile of long COVID symptoms needing rehabilitation: a cross-sectional household survey of 12,925 SARS-CoV-2 cases between July and December 2021 in Bangladesh

**DOI:** 10.1186/s13690-023-01140-0

**Published:** 2023-07-17

**Authors:** Md. Feroz Kabir, Khin Nyein Yin, Mohammad Saffree Jeffree, Fatimah Binti Ahmedy, Sharmila Jahan, Md. Waliul Islam, Iqbal Kabir Jahid, Manoj Sivan, Sonjit Kumar Chakrovorty, K. M. Amran Hossain

**Affiliations:** 1 Faculty of Medicine and Health Sciences, Universiti Malaysia Sabah, Kota Kinabalu, Sabah, 88400 Malaysia; 2 Department of Physiotherapy and Rehabilitation, Jashore University of Science and Technology (JUST), Jashore, Bangladesh; 3 Department of Physiotherapy, Centre for the Rehabilitation of the Paralysed (CRP), Savar, Dhaka Bangladesh; 4 Department of Microbiology, Jashore University of Science and Technology (JUST), Jashore, Bangladesh; 5 Leeds Institute of Rheumatic and Musculoskeletal Medicine, School of Medicine, Faculty of Medicine and Health, University of Leeds, Leeds, UK

**Keywords:** Long COVID, Epidemiology, C19-YRS, Rehabilitation, Bangladesh

## Abstract

**Background and aims:**

: It is important to determine the profile of long COVID (LC) symptoms within the scope of rehabilitation in Bangladesh. This study’s objective was to estimate the newly experienced long COVID symptoms needing rehabilitation by determining the prevalence and spectrum of impairments due to LC in Bangladesh.

**Methods:**

A Cross-sectional household survey of 12,925 COVID-19 patients confirmed by RT-PCR from 24 testing facilities in Bangladesh. LC was diagnosed according to WHO working group definition. COVID-19 Yorkshire Rehabilitation Scale (C19-YRS) was used to determine the symptom responses, symptom severity, new long COVID symptoms, and scope of rehabilitation.

**Results:**

The population proportion of LC symptoms requiring rehabilitation interventions are 0.22 [*95% CI, 0.20–0.24*] in Bangladeshi people diagnosed with SARS-CoV-2. Among them, *0.08 [95% CI, 0.07–0.09]* had mild, *0.07 [95% CI, 0.06–0.09]* had moderate, and *0.05 [95% CI, 0.04–0.06]* had severe long COVID symptoms (LCS). There was a significant positive correlation between LCS and functional disabilities (r = 0.889, p < 0.001), while a negative correlation was observed between the severity of symptoms and overall health (r=-0.658, p < 0.001). In comparison to the pre-COVID status, 17 new LCS were observed and the increase in the scope of rehabilitation intervention among LCS ranged between *0.01 [95% CI, 0.001–0.01]* and *0.21 [95% CI, 0.19–0.22]*. In Bangladesh, 59% (n = 334) of the LC cases are out of reach for any rehabilitation interventions.

**Conclusion:**

Nearly one-fourth of Bangladeshi Post-COVID-19 have long COVID (LC). Seventeen symptoms (LCS) were observed and more than half of the populations having long COVID are out of reach of any rehabilitation facilities.

**Table:** 

**Text box 1. Contributions to the literature**
• Research has shown that the sequel of COVID-19 has long-term persistent symptoms and the spectrum of impairments or episodic disabilities is invisible.
• Long COVID certainly has a bio-psychosocial impact and rehabilitation is the best possible approach for the management of long COVID. For many reasons, the scope of rehabilitation for long COVID is yet to be determined in lower middle-income nations.
• This paper adds the population proportion of long COVID along with the determination of the scope of rehabilitation for long COVID in Bangladesh. Moreover, this paper also predicts the out-of-reach population for long COVID rehabilitation in Bangladesh.

## Introduction

Long COVID or post-COVID-19 condition is persistent symptoms irrelevant to any other diagnosis, which can last for at least two months in people with SARS-COV-2 infection after 3 months from the onset [1]. The global prevalence of Long COVID (LC) survivors varies between 10 to 36% [2]. A household survey reported the prevalence of LC in Bangladesh was 16.1% as of 2020 [3] and the rate increased to 25.2% in another survey [4]. A variety of symptoms were reported in both studies with major symptoms which included fatigue, musculoskeletal pain, headache, loss of concentration, anxiety, depression, and post-exertion dyspnea [3, 4]. LC is not only a sequel of COVID-19, but also it has an unpredictable disease course that is evident to be extended to more than a year, and a variety of symptoms are prevalent with the changing of time [5]. The management of LC is multidisciplinary as the condition impacts multiple organs in humans [6]. Rehabilitation is the key to the recovery of persistent illness which extends for a long time and interferes with body function, daily activities, and overall quality of life [7].

A recent study found that Bangladeshi LC has some significant impairments in the musculoskeletal, neurological, and cognitive domains [4], but the overall scope of rehabilitation was not examined. The published studies addressed the prevalence but there are gaps in population proportion. Published studies used the COVID-19 Yorkshire rehabilitation scale to measure symptom response, functional impairments, and disability [8]. Using this tool, a study in the UK screened 370 post-COVID cases and presented three types of severity; mild (n = 90), moderate (n = 186), and severe (n = 94) [9]. The study elicited 12 major long COVID symptoms (LCS) in the respondents with complaints of fatigue (95%), musculoskeletal and body pain (89%), anxiety (89%), breathlessness (85%), and cognitive difficulties (85%) [9]. A recent study found that returning to usual physical activities is the major challenge for people with LCS as it worsens the symptoms [10]. The stated study recommended finding out the best possible approach to restore the physical functioning of individuals to prevent disability for LCS.

The World health organization (WHO) stated that rehabilitation is an integral part of universal health and well-being [11]. The global scope of rehabilitation stands for 2.4 billion people and 50% of the people living in lower-middle-income countries are out of reach of the scope [11]. COVID-19 changed the paradigm of rehabilitation service by adding a set of new impairments within the spectrum of rehabilitation [9]. As of December 31, 2021, Bangladesh has 1,585,539 confirmed COVID-19 cases where approximately 253,686 cases suffering from LCS as the prevalence study (16.1%) in Bangladesh [3]. Determination of the scope of LC rehabilitation is a necessity to implement the comprehensive rehabilitation program. The aim of this study is (1) to estimate the population proportion of long COVID symptoms (LCS) in Bangladesh (2) to investigate the relationship between the spectrum of symptoms and functional impairments, and (3) to determine the status of LCS receiving rehabilitation services.

## Methodology

### Study design, samples, and sampling method

**Fig. 1:**
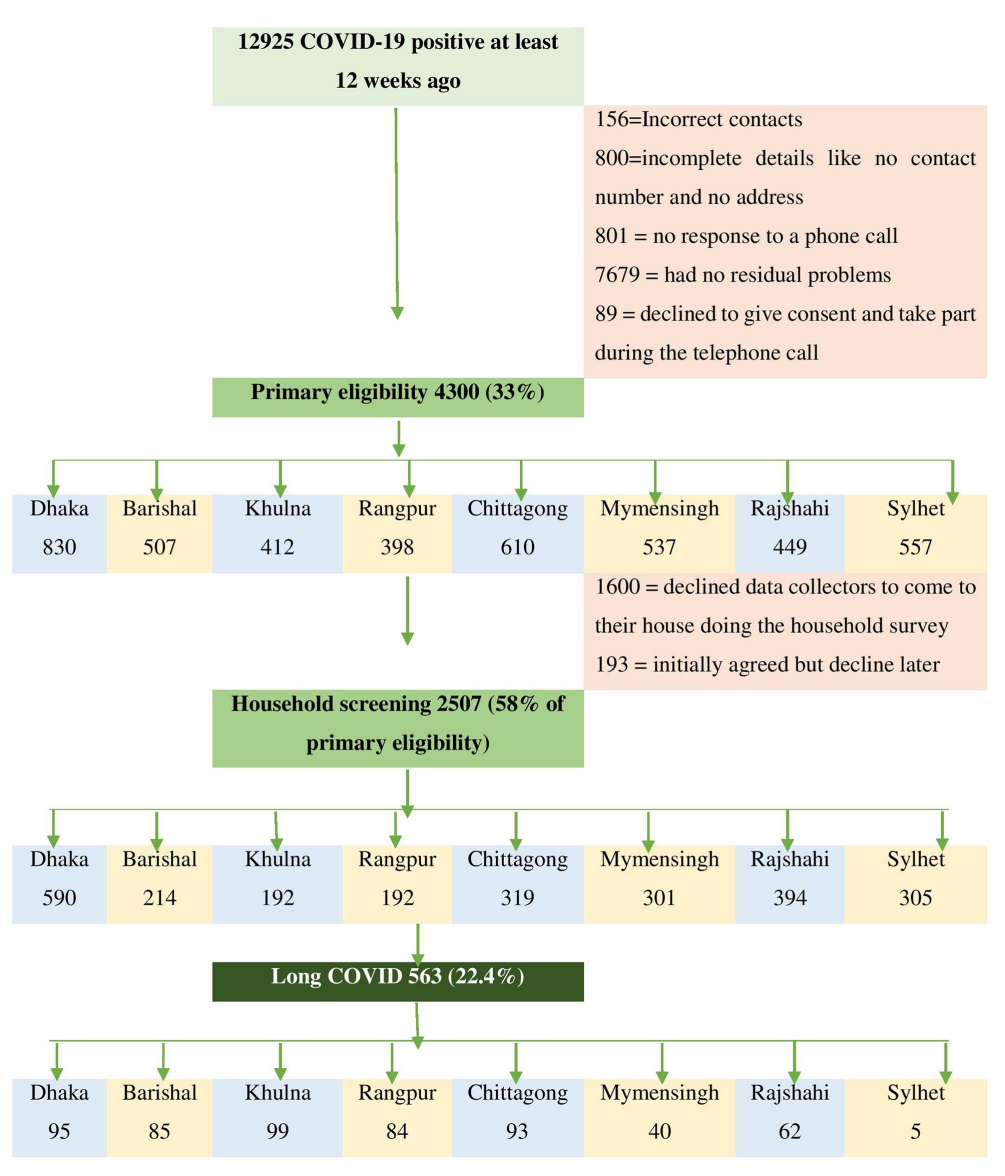
Process of epidemiological household screening

This study was a cross-sectional household survey of COVID-19 survivors with COVID-19 positive in a real-time polymerase chain reaction test (RTPCR) at 24 Bangladeshi COVID testing centers between July and December 2021. The population frame was 12,925 COVID-19-positive patients listed with a test date at least 12 weeks ago. There were about 4300 out of 12,925 participants who met primary eligibility criteria which were aged 18 years and above, responded to the telephone call, had persistent symptoms, and provided consent for a household survey. Among 4300, 2507 people agreed to a face-to-face interview (Fig. 1). The data was collected by the trained data collector’s in-person and the cases were confirmed as long COVID according to the diagnosis guideline [1]. The sampling process was stratified random sampling and our strata were the eight administrative divisions of Bangladesh. The sample size was estimated with a 95% confidence interval and a 1.0 design efficacy with a 5% margin of error resulting in 1,800 for this study. The minimum number of samples to be collected per strata was 190. The process was performed by Epi Info version 7.2.0.

### Study procedure

The study followed the Strengthening the Reporting of Observational Studies in Epidemiology (STROBE) guideline for a cross-sectional study. Figure 1 shows the flow chart of the study process. Twelve final-year bachelor’s of physiotherapy students volunteered as data collectors of the study. They were trained to collect the household data through pretested and validated questionnaires including the COVID-19 Yorkshire rehabilitation scale (C19-YRS).

### Tools

Self-administrated socio-demographic, COVID-19 clinical characteristics questionnaire and COVID-19 Yorkshire Rehabilitation Scale (C19-YRS) were used to determine the symptoms and functional disability and the scope of rehabilitation based on symptoms, functional disability, and the number of patients with long COVID. C19 YRS is a valid and reliable tool for symptom responses, symptom severity, and determining the scope of rehabilitation for post-COVID-19 symptoms [8]. The C19-YRS has 22 items with each item rated on an 11-point numerical rating scale from 0 (none of this symptom) to 10 (extremely severe level or impact) experienced by the person before and after COVID-19. The questionnaire is further subcategorized into four subscales as symptom severity score (0–100), functional disability score (0–50), additional symptoms (0–60), and overall health (0–10). Thus, we can easily determine the noble symptoms, symptom spectrum, and disease course. The original questionnaire was in English, and it was translated into Bangla. A forward and backward translation was performed to ensure the standards of internal consistency of the questionnaire (Cronbach alpha score 0.879). From the 12,925-sample frame, 33% (n = 4300) were primarily eligible for screening and invited to take part in the study, the response rate was 58% (n = 2507).

### Data analysis

After completing the initial data collection, each questionnaire was double-checked for any errors or ambiguous information. SPSS version 25 was used to analyze the data. In a list, the variables were labeled in sequence. The researcher named the variables and established the types, values, decimals, label alignment, and measurement level of data in the SPSS variable view. The data was then double-checked to ensure that all the information from the questionnaire had been sent. The variables were determined as nominal, ordinal, interval, and ratio data and considered their parametric or non-parametric properties based on the data type, normality test, and standard procedure. One-way ANOVA and Friedman’s ANOVA tests were used to determine the significant impact of socio-demographic characteristics on the mild, moderate, and severe symptoms. Kolmogorov Smirnov test was performed for symptom severity score, functional disability score, and overall health score. Descriptive statistics were used to analyze the demographic distribution of long COVID and the cases with no long COVID symptoms and the mean severity score of symptoms, functioning, and disability in long COVID-19. Population proportion has been calculated through binominal “exact” calculation with 95% CI. The mean symptom score was used to determine the overall intensity of the 17 most reported symptoms, with a score of 6 or more being considered “severe,“ 3 to 5.9 “moderate,“ and less than 3 “Mild” [9]. Severity also visually represented by a radar plot. A heat plot represents the correlation between symptoms and functional limitations. The correlation among C19-YRS symptom severity score, functional disability score, and overall health score was determined through Pearson correlation. The alpha value was set as P < 0.05.

## Results

### Population proportion of long COVID symptoms (LCS) in Bangladesh

As of 4th November 2022, there were 2,035,745 confirmed COVID-19 cases and 29,425 deaths in Bangladesh hence 2,006,320 COVID-19 cases survived [12]. According to our study, the long COVID prevalence was 0.22 [*95% CI, 0.20–0.24*] and we estimated that there were 449,415 long COVID cases among COVID survivors in Bangladesh till 4th November 2022.

### Socio-demographic profile of long COVID

**Fig. 2:**
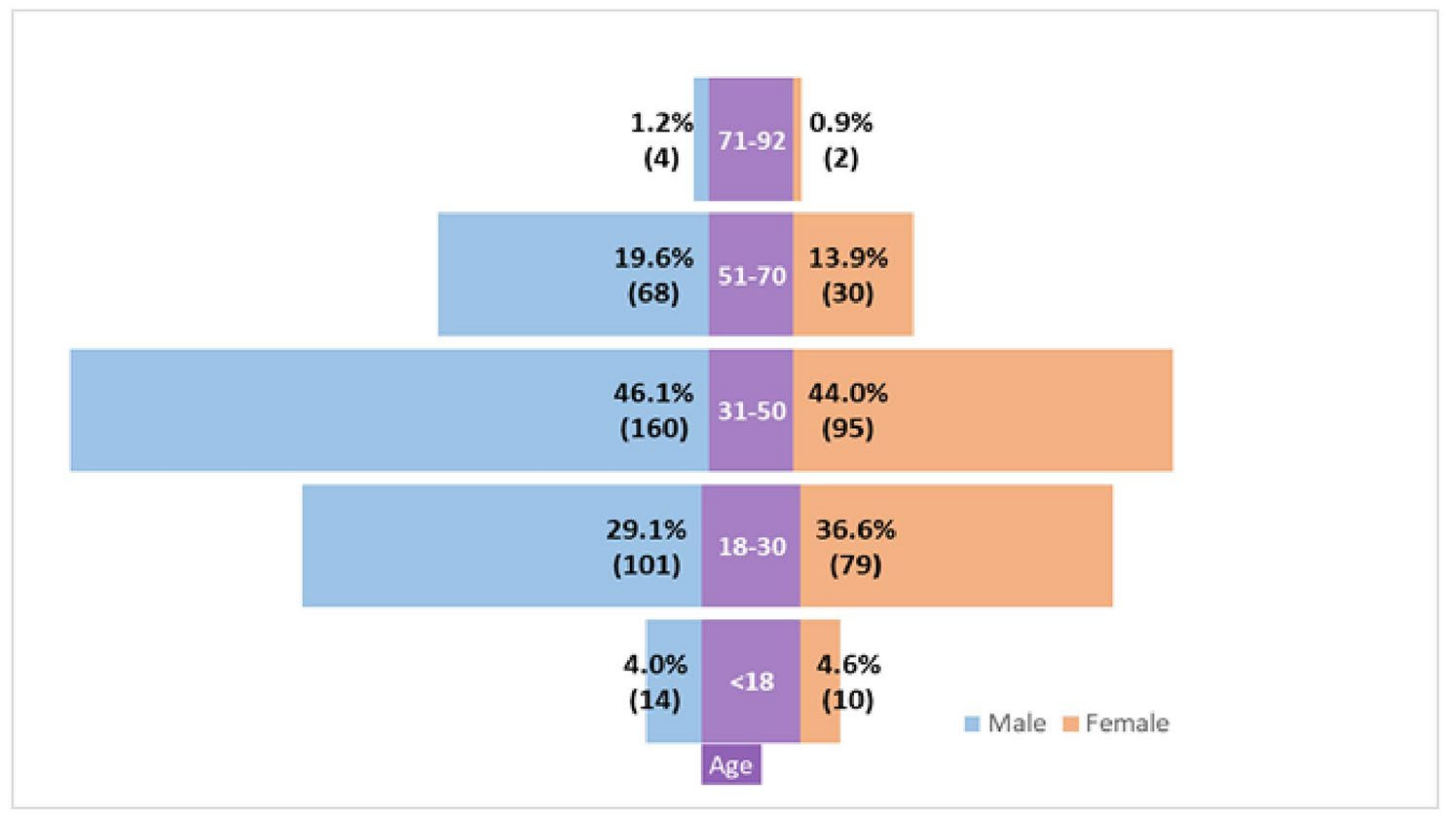
Gender of Long COVID respondents according to age

**Fig. 3:**
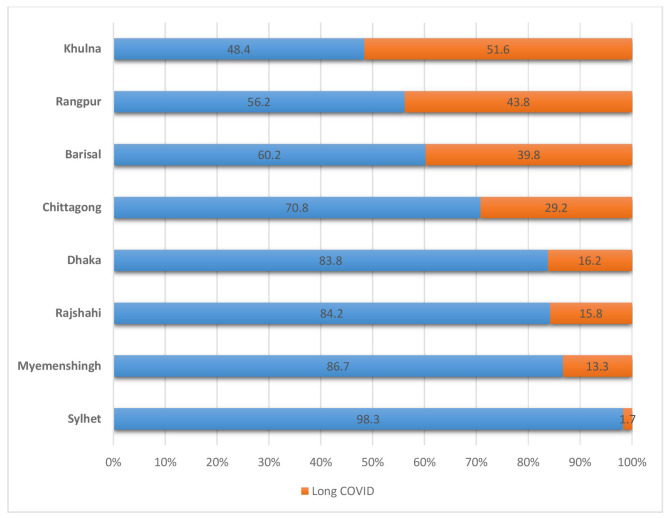
Demographic distribution of long COVID and the cases with no long COVID symptoms in Bangladesh

Table 1 shows the socio-demographic profile. The mean age of the respondents was 35.04 ± 9.1 years and the male-female ratio was 1.94:1. Among the long COVID survivors, 11% of the respondents (severe case) were hospitalized during COVID-19 infection, and 89% were non-hospitalized so they had mild to moderate COVID-19. At least one family member with a COVID-19 diagnosis was found in 44% of the respondents and 64% had at least one diagnosis in the community (ward or village). Long COVID cases ratio in semi-urban, urban, and rural was 5:3:1. 96% of the long COVID cases were vaccinated (1st dose 11%, 2nd dose 59%, and booster dose 26%). Among the long COVID survivors, the distribution of severity was mild (38.97%) moderate (35.23%), and severe (25.80%). Figure 2 shows that middle-aged people both male and female were more affected with long COVID rather than children and older people. About 46.10% (n = 160) male and 44% (n = 95) female participants had long COVID of 31–50 years of age. Figure 3 reveals the highest percentage of long COVID 51.6% in Khulna and the lowest percentage 1.7% in Sylhet. The second height% of long COVID 43.8% was in the Rangpur division.

**Table 1:** Long COVID distribution according to demography surveyed on 12,925 SARS-CoV-2 cases between July and December 2021 in Bangladesh

Variables	Sub-category	ALL (N = 2507)		No long COVID symptoms (N = 1944)		Long COVID (N = 563)		Mild (N = 220; 0.08 [0.07-0.09]		Moderate (N = 198; 0.07 [0.06-0.09]		Severe (N = 145; 0.05 [0.04-0.06]		P
**Age** ^**a**^	Mean ± SD	37	± 13.9	37	± 14	**37.2**	**± 13.7**	37.5	± 13.7	36.8	± 13.3	37.4	± 14.5	0.930
**Gender** ^**b**^	Male	1530	(61%)	1183	(61%)	**347**	**(62%)**	145	0.05 [0.04-0.06]	118	0.04 [0.03-0.05]	83	0.03 [0.02-0.04]	0.223
	Female	977	(39%)	761	(39%)	**216**	**(38%)**	75	0.02 [0.02-0.03]	80	0.03 [0.02-0.03]	62	0.02 [0.01-0.03]	
**Hospitalization** ^**b**^	Hospitalized	280	(11.17%)	217	(11.16%)	**63**	**(11.20%)**	26	0.01 [0.00-0.01]	22	0.008 [0.00-0.01]	15	0.006 [0.00-0.009]	0.0001***
**At least one COVID case in community** ^**b**^	Yes	1615	(64%)	1284	(66%)	**331**	**(59%)**	119	0.04 [0.03-0.05]	113	0.04 [0.03-0.05]	98	0.03 [0.03-0.04]	0.0001***
**At least one COVID case in family** ^**b**^	Yes	1100	(44%)	871	(45%)	**229**	**(41%)**	88	0.03 [0.02-0.04]	83	0.03 [0.02-0.04]	58	0.02 [0.01-0.02]	0.0001***
**Vaccination** ^**b**^	Non-Vaccinated	109	(6%)	87	(4%)	**22**	**(4%)**	9	0.003 [0.00-0.006]	9	0.003 [0.00-0.006]	4	0.001 [0.00-0.004]	0.0001***
	1st dose	259	(13%)	196	(10%)	**63**	**(11%)**	22	0.008 [0.00-0.01]	20	0.008 [0.00-0.01]	21	0.008 [0.00-0.01]	
	2nd dose	1589	(82%)	1256	(65%)	**333**	**(59%)**	124	0.04 [0.04-0.05]	125	0.04 [0.04-0.05]	84	0.03 [0.02-0.04]	
	Booster dose	550	(28%)	405	(21%)	**145**	**(26%)**	43	0.01 [0.01-0.02]	65	0.02 [0.02-0.03]	36	0.01 [0.01-0.02]	
**Living area** ^**b**^	Urban	228	(9%)	168	(9%)	**60**	**(11%)**	29	0.01 [0.00-0.01]	18	0.007 [0.00-0.01]	13	0.005 [0.00-0.008]	0.324
	Rural	848	(34%)	651	(33%)	**197**	**(35%)**	93	0.03 [0.03-0.04]	62	0.02 [0.01-0.03]	42	0.01 [0.01-0.02]	
	Semi urban	1431	(57%)	1125	(58%)	**306**	**(54%)**	97	0.03 [0.03-0.04]	118	0.04 [0.03-0.05]	90	0.03 [0.02-0.04]	
**Attending Rehabilitation** ^**b**^	Yes	237	(9%)	8	(0.4%)	**229**	**(41%)**	52	0.02 [0.01-0.02]	100	0.03 [0.03-0.04]	77	0.03 [0.02-0.03]	0.0001***

^a^One-way ANOVA, ^b^Friedman’s ANOVA among mild, moderate, and severe cases with significant values (P) as < 0.001***, < 0.01**, and < 0.05*

### Long COVID symptoms within the scope of rehabilitation

**Fig. 4:**
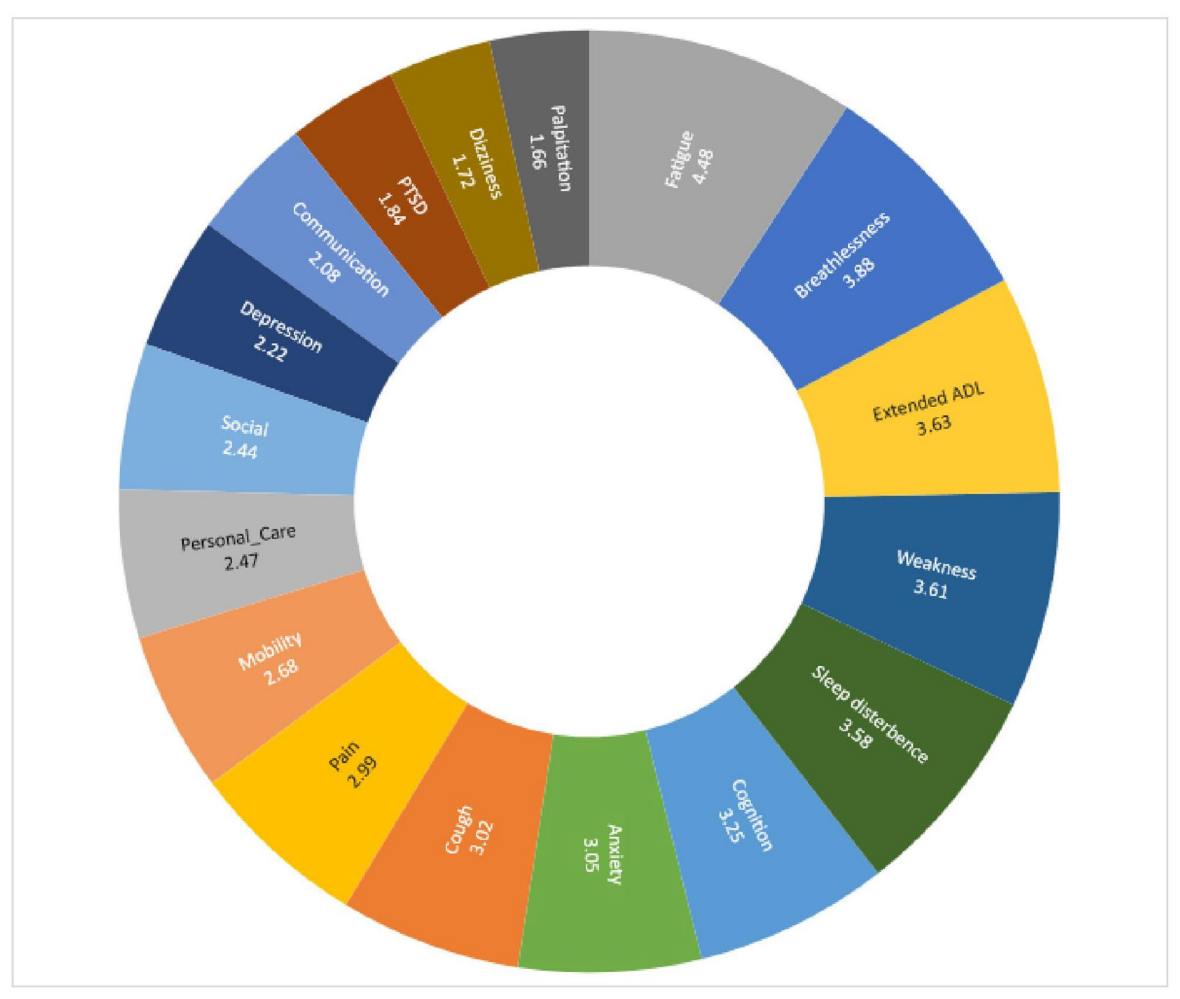
Mean severity score of symptoms, functioning, and disability in long COVID-19

**Fig. 5:**
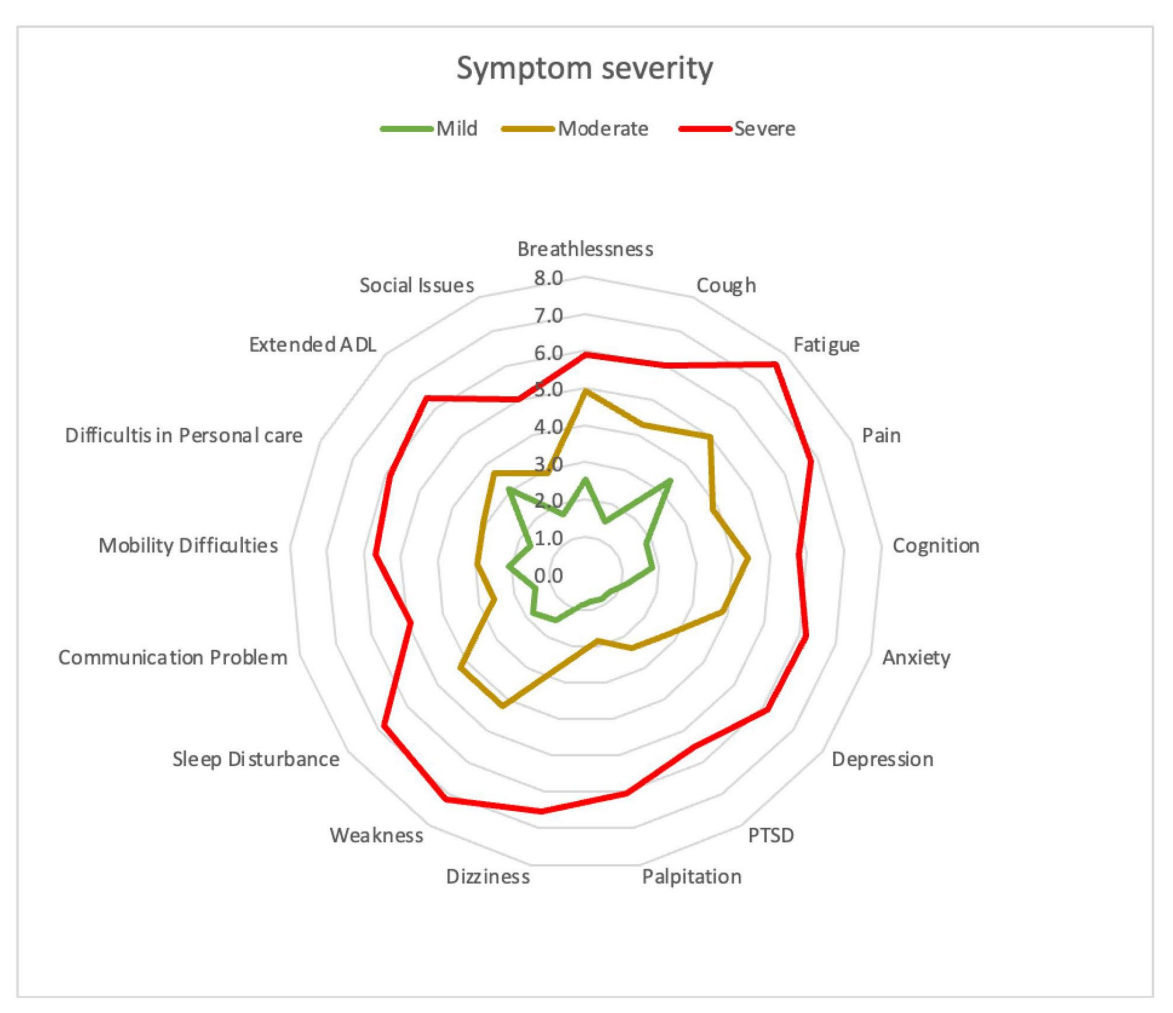
Radar plot of mean symptom score according to the severity

The prolonged generalized weakness was the most common symptom experienced with 0.21 [*95% CI, 0.19–0.22*], followed by fatigue at 0.18 [*95% CI, 0.17–0.20*], sleep disturbance at 0.16 [*95% CI, 0.14–0.17*], cough 0.14 [*95% CI, 0.13–0.16*], muscle pain 0.14 [*95% CI, 0.13–0.16*], arthralgia 0.12 [*95% CI, 0.11–0.13*] anxiety and depression 0.12 [*95% CI, 0.10–0.13*], problem in playing social role 0.12 [*95% CI, 0.10–0.13*], headache 0.11 [*95% CI, 0.10–0.12*], problem in daily living activities 0.10 [*95% CI, 0.08–0.11*], and memory problem 0.09 [*95% CI, 0.08–0.10*]. Table 2 shows 30 new LCS, among them, 17 require long COVID rehabilitation services. The mean severity score of symptoms and functional disability measured by the COVID-19 Yorkshire Rehabilitation 0–10 Scale (C19-YRS) was illustrated in Fig. 4. A radar plot illustrated the mean symptom score for mild, moderate, and severe categories (Fig. 5). It shows that there was a gradient in all 17 symptom scores presented according to the highest values in mean with patients who had greater overall symptoms having each separate symptom higher on average with no single symptom driving this association. The average score means that fatigue, pain, and weakness are similar in the severe category, but for the mild category, the average score of fatigue is much higher than weakness. The mean symptom score according to the severity was measured by COVID-19 Yorkshire Rehabilitation 0–10 Scale (C19-YRS).

**Table 2:** Population Proportion of new Long COVID symptoms

Symptoms	Before COVID-19, n (%)	12 weeks after COVID-19, n (%)	Long COVID cases, n out of N = 2507	Proportion of new Long COVID cases, [*95% CI]*
Prolonged Generalized Weakness	0	788; 31.4%	533	0.21 [0.19-0.22]
Fatigue	132; 5.3%	765; 30.5%	475	0.18 [0.17-0.20]
Sleep problems or disturbance	0	407; 16.2%	407	0.16 [0.14-0.17]
Cough	24; 1%	398; 15.9%	374	0.14 [0.13-0.16]
Muscle pain	47; 1.9%	419; 16.7%	372	0.14 [0.13-0.16]
General Arthralgia	44; 1.8%	359; 14.3%	315	0.12 [0.11-0.13]
Anxiety based on the YRS scale	68; 2.7%	375; 15%	307	0.12 [0.10-0.13]
Depression based on the YRS scale	67; 2.7%	373; 14.9%	306	0.12 [0.10-0.13]
The problem with playing a social role	61; 2.4%	364; 14.5%	303	0.12 [0.10-0.13]
Headache	19; 0.8%	308; 12.3%	289	0.11 [0.10-0.12]
The problem in Activities of Daily Living	48; 1.9%	301; 12%	253	0.10 [0.08-0.11]
Dizziness	0	272; 10.8%	272	0.10 [0.09-0.12]
Prolonged Fever	0	246; 9.8%	246	0.09 [0.08-0.11]
Memory problem	28; 1.1%	257; 10.3%	229	0.09 [0.08-0.10]
Problem with personal care	38; 1.5%	265; 10.6%	227	0.09 [0.07-0.10]
Problem in Concentration	34; 1.4%	250; 10%	216	0.08 [0.07-0.09]
Breathlessness or Dyspnea	116; 4.6%	321; 12.8%	205	0.08 [0.07-0.09]
Mobility problem	41; 1.6%	238; 9.5%	197	0.07 [0.06-0.08]
Palpitation	0	150; 6%	150	0.05 [0.05-0.06]
Chest pain	11; 0.4%	104; 4.1%	93	0.03 [0.03-0.04]
Skin Rash	0	66; 2.6%	66	0.02 [0.02-0.03]
Swallowing and feeding problem	0	30; 1.2%	30	0.01 [0.008-0.01]
Communication problem	14; 0.6%	42; 1.7%	28	0.01 [0.007-0.01]
Voice change	6; 0.2%	21; 0.8%	15	0.006 [0.003-0.009]
Problem with executing a plan	6; 0.2%	22; 0.9%	16	0.006 [0.003-0.01]
PTSD based on the YRS scale	4; 0.2%	20; 0.8%	16	0.006 [0.003-0.01]
Abdominal Pain	6; 0.2%	20; 0.8%	14	0.005 [0.003-0.009]
Noisy breathing	6; 0.2%	14; 0.6%	8	0.003 [0.001-0.006]
Bladder control problem	5; 0.2%	7; 0.3%	2	0.001 [0.0001-0.002]
Bowel control problem	4; 0.2%	5; 0.2%	1	0.001 [0.00001-0.002]

Long COVID defined according to WHO working group clinical criteria;

C19-YRS, COVID-19 Yorkshire Rehabilitation Scale; PTSD, Post-Traumatic Stress Disorder;

### Relationship among long COVID symptoms and impairments

**Fig. 6:**
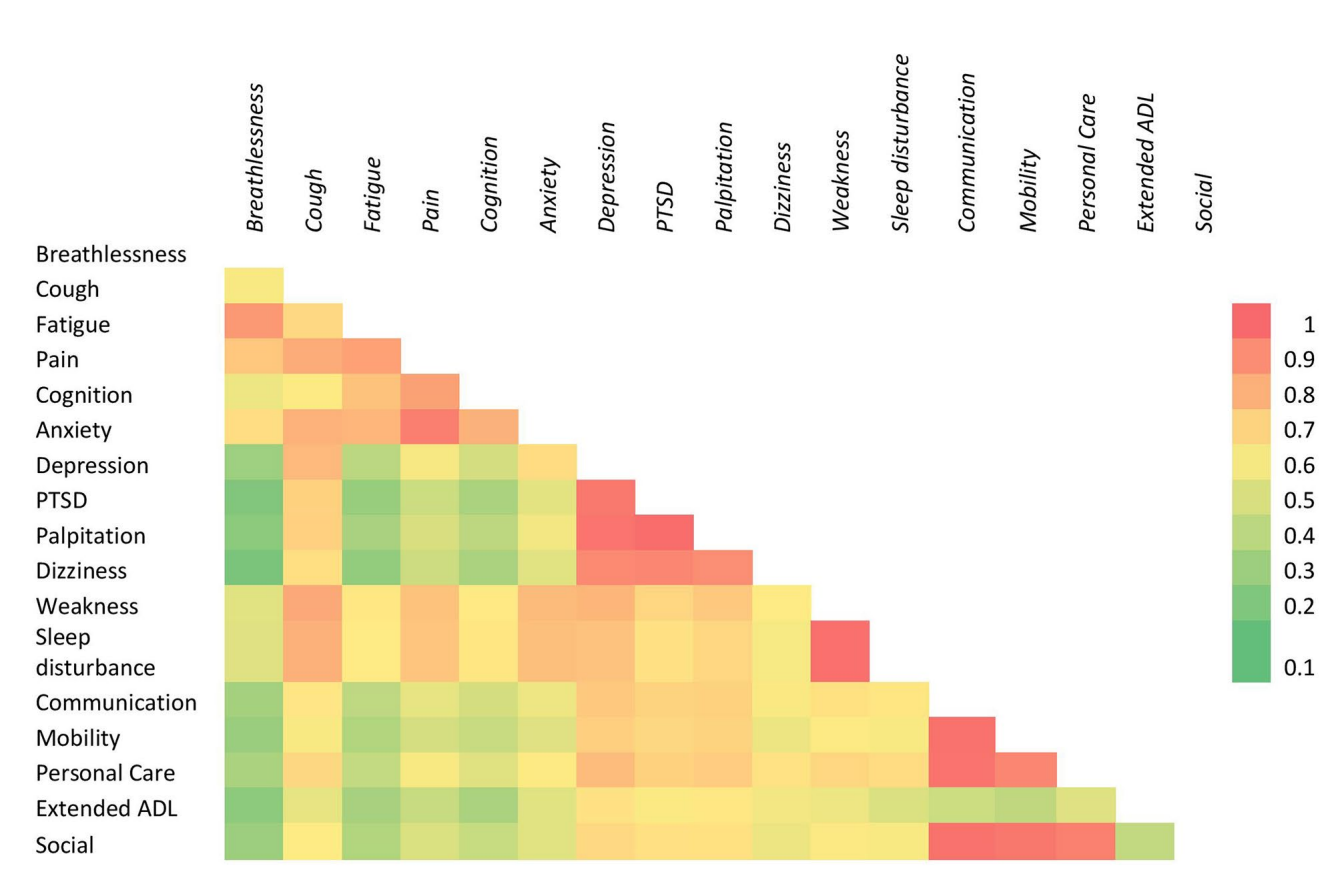
Relationship among the spectrum of Long COVID symptoms

Figure 6 represents the heat map of symptoms according to the Pearson correlation of symptom severity scores. Strong association > 0.7 was marked as red. There was a positive and linear correlation between fatigue and breathlessness, pain and fatigue, cognition and anxiety, depression and dizziness, PTSD, and palpitation, the lack of sleep and exertion or weakness, mobility and communication, personal care, and social participation. A strong linear and positive correlation was found between symptom severity in the C19-YRS subscale and functional disability subscale (r = 0.889, P < 0.001). A reverse and strong correlation of overall health was found with symptom severity (r=-0.65, P < 0.001) and functional disability (r=-0.64, P < 0.001). The mean severity score of symptoms and functional disability measured by the COVID-19 Yorkshire Rehabilitation 0–10 Scale (C19-YRS) was illustrated in Fig. 4. A radar plot illustrated the mean symptom score for mild, moderate, and severe categories (Fig. 5). It shows that there was a gradient in all 17 symptom scores presented according to the highest values in mean with patients who had greater overall symptoms having each separate symptom higher on average with no single symptom driving this association. The average score means that fatigue, pain, and weakness are similar in the severe category, but for the mild category, the average score of fatigue is much higher than weakness. The mean symptom score according to the severity was measured by COVID-19 Yorkshire Rehabilitation 0–10 Scale (C19-YRS).

### Status of long COVID cases attending rehabilitation service

41% (n = 229) out of total long COVID cases (n = 563) attended rehabilitation services in eight administrative divisions in Bangladesh. 24% (n = 52) of mild long COVID cases, 51% (n = 100) of moderate long COVID patients, and 53% (n = 77) of severe long COVID patients (Table 1) attended rehabilitation services. The rate of increasing new cases requiring rehabilitation ranged between 0.01 *[95% CI, 0.001–0.01]* and 0.21 *[95% CI, 0.19–0.22].*

## Discussion

The study result addressed all the objectives including the prevalence of long COVID and the scope of rehabilitation for long COVID in Bangladesh. The study also presented a variety of symptoms and impairments based on severity, spectrum, and correlation among the various symptoms. Furthermore, the study presented that more than half of the stakeholders who had moderate and severe long COVID attended rehabilitation centers in Bangladesh. The study fills up the research gap to estimate the new long COVID cases according to symptoms and determine the scope of rehabilitation.

The study presented the prevalence of long COVID as 0.22 [*95% CI, 0.20–0.24*]. Another cohort conducted in 2020 stated the prevalence as 16.1% [3] before the delta variant induction [13] in Bangladesh. The Prevalence of long COVID with delta variant in 2021 was 25.2% [4]. A recent meta-analysis found the global prevalence of post-COVID symptoms after 90 days (long COVID) as 0.32 *[95% CI: 0.14, 0.57].*[14]. Our study period was in the last phase of delta community transmission and before the introduction of the omicron variant in Bangladesh [15]. The prevalence of long COVID after 28 days of diagnosis in north India was 28.2% in 2020 [16], and 42% reported persistent symptoms within 1–3 months of diagnosis of COVID-19 in Pakistan in 2021 [17]. This study showed that the prevalence of long COVID cases in Bangladesh increased with time. Our study found significant variations of prevalence in different divisions of Bangladesh. Khulna, the neighboring division of the delta epicenter of India had the highest percentage of long COVID 51.6%, and Sylhet had the lowest percentage 1.7%. The variation is consistent with previous cohort designs with a household survey [3, 4]. The population pyramid of Bangladesh states by gender and age group that 0–14 years (children), 15–24 years (early working age), 25–54 years (prime working age), 55–64 years (mature working age), 65 years and over (elderly) [18]. Our study found that most long COVID patients were early working age and prime working age or middle-aged, male in gender, and with moderate and severe categories. WHO reported [19] that 85% of the long COVID cases in the USA had mild COVID-19 disease, and long COVID is mostly prevalent in middle age. The study suggested that one-third of the adults had new or persistent post-COVID symptoms three weeks after COVID-19 [20].

Our study found 17 major symptoms that explore the scope of comprehensive rehabilitation within mixed domains 89% musculoskeletal domain, 87% cardio-respiratory domain, 83% cognitive and neurological domain, 91% mental health domain, 97% in the functional and social participation domain. This proved that the symptoms are interrelated, and the impairments magnify the association of symptoms as clusters. Among the new long COVID cases, the rapidly growing symptoms requiring rehabilitation were prolonged generalized weakness, fatigue, muscle pain, joint pain, anxiety and depression, problem in social life, problem in daily living activities, memory problem, problems in personal care, and problems in mobility. The reason behind this is the multisystem involvement of COVID-19 causing a spectrum of post-COVID symptoms [1, 2, 21]. Evidence suggested managing long COVID with a multidisciplinary approach since the impairments are multidirectional and significantly have a long-term impact on the human lifespan [22]. Our study found a positive and linear correlation among individual symptoms based on C19-YRS. Evidence suggested that the overall perception of health and patients’ reports of symptoms, functioning, and disability have a positive relationship [23]. The symptom severity and functional limitations harm the overall health of long COVID survivors. These impairments require safe long COVID rehabilitation management [24] by a comprehensive skilled multidisciplinary team. Out of 41% of long COVID cases in this study attended rehabilitation services. More than half of the Long COVID patients didn’t receive the rehabilitation service. Therefore, there is an emerging necessity for developing an integrated long COVID care and rehabilitation pathway [25] in the healthcare system in Bangladesh. The new report stated that there were about 2,712 long COVID patients referred to the rehabilitation center in Hospital Sungai Buloh, Malaysia from November 2021 to September 2022 [26].

### Strength and limitations

The strength of this study was following STROBE guidelines, adhering to ethical practice, randomization of samples, and household screening of symptoms. There were some limitations, but they did not affect the study results significantly. The responses from different strata were uneven, due to the uneven testing rate and response rate across the divisions of Bangladesh. Therefore, discrepancies in long COVID prevalence among divisions were seen. This was believed to not affect the population estimation of long COVID prevalence in Bangladesh. The study addressed the existing research gap on the scope of rehabilitation and current perspectives of integrated long COVID management in Bangladesh. Our study predicted 449,415 long COVID cases among RT PCR-positive cases. However, there are no official long COVID rehabilitation centers in Bangladesh. Hence, the recommendations from this study measure the status of the long COVID rehabilitation workforce and facilities, clinical cohorts of long COVID symptoms, and clinical trials in the scope of rehabilitation.

## Conclusion

In Bangladesh, the prevalence of LCS needing rehabilitation was 0.22 [*95% CI, 0.20–0.24*] for COVID-19 cases diagnosed from July to December 2021. There is an increased necessity for rehabilitation services, as the rate of new symptoms observed between *0.01 [95% CI, 0.001–0.01]* and *0.21 [95% CI, 0.19–0.22]* of total LCS cases. 59% of the LCS cases are out of reach from any long COVID rehabilitation services.

## Data Availability

The data is available upon request to the first author at feroz@just.edu.bd.

## Abbreviations

LC: Long COVID
LCS: Long COVID symptoms
DGHS: Directorate General of health services
CTRI: Clinical trial registry India
C19YRS: COVID-19 Yorkshire Rehabilitation Scale
